# *Leishmania amazonensis* Subverts the Transcription Factor Landscape in Dendritic Cells to Avoid Inflammasome Activation and Stall Maturation

**DOI:** 10.3389/fimmu.2020.01098

**Published:** 2020-06-09

**Authors:** Hervé Lecoeur, Thibault Rosazza, Kossiwa Kokou, Hugo Varet, Jean-Yves Coppée, Arezou Lari, Pierre-Henri Commère, Robert Weil, Guangxun Meng, Genevieve Milon, Gerald F. Späth, Eric Prina

**Affiliations:** ^1^Institut Pasteur, INSERM U1201, Unité de Parasitologie Moléculaire et Signalisation, Département des Parasites et Insectes Vecteurs, Paris, France; ^2^Pasteur Institute of Shanghai, Innate Immunity Unit, Key Laboratory of Molecular Virology and Immunology, Shanghai Institutes for Biological Sciences, Chinese Academy of Sciences, Shanghai, China; ^3^Pasteur International Unit “Inflammation and Leishmania Infection”, Paris, France; ^4^Hub de Bioinformatique et Biostatistique – Département Biologie Computationnelle, Institut Pasteur, USR 3756 CNRS, Paris, France; ^5^Institut Pasteur - Transcriptome and Epigenome Platform - Biomics Pole - C2RT, Paris, France; ^6^Systems Biomedicine Unit, Institut Pasteur of Iran, Teheran, Iran; ^7^Institut Pasteur, Plate-Forme de Cytométrie, Paris, France; ^8^Sorbonne Universités, Institut National de la Santé et de la Recherche Médicale (Inserm, UMR1135), Centre National de la Recherche Scientifique (CNRS, ERL8255), Centre d'Immunologie et des Maladies Infectieuses CIMI, Paris, France; ^9^Institut Pasteur, Laboratoire Immunophysiologie et Parasitisme, Département des Parasites et Insectes Vecteurs, Paris, France

**Keywords:** dendritic cell, *Leishmania amazonensis*, amastigote, transcription factor, NF-κB, NLRP3, transcriptome, cell sorting

## Abstract

*Leishmania* parasites are the causative agents of human leishmaniases. They infect professional phagocytes of their mammalian hosts, including dendritic cells (DCs) that are essential for the initiation of adaptive immune responses. These immune functions strictly depend on the DC's capacity to differentiate from immature, antigen-capturing cells to mature, antigen-presenting cells—a process accompanied by profound changes in cellular phenotype and expression profile. Only little is known on how intracellular *Leishmania* affects this important process and DC transcriptional regulation. Here, we investigate these important open questions analyzing phenotypic, cytokine profile and transcriptomic changes in murine, immature bone marrow-derived DCs (iBMDCs) infected with antibody-opsonized and non-opsonized *Leishmania amazonensis* (*L.am*) amastigotes. DCs infected by non-opsonized amastigotes remained phenotypically immature whereas those infected by opsonized parasites displayed a semi-mature phenotype. The low frequency of infected DCs in culture led us to use *Ds*Red2-transgenic parasites allowing for the enrichment of infected BMDCs by FACS. Sorted infected DCs were then subjected to transcriptomic analyses using Affymetrix GeneChip technology. Independent of parasite opsonization, *Leishmania* infection induced expression of genes related to key DC processes involved in MHC Class I-restricted antigen presentation and alternative NF-κB activation. DCs infected by non-opsonized parasites maintained an immature phenotype and showed a small but significant down-regulation of gene expression related to pro-inflammatory TLR signaling, the canonical NF-kB pathway and the NLRP3 inflammasome. This transcriptomic profile was further enhanced in DCs infected with opsonized parasites that displayed a semi-mature phenotype despite absence of inflammasome activation. This paradoxical DC phenotype represents a *Leishmania*-specific signature, which to our knowledge has not been observed with other opsonized infectious agents. In conclusion, systems-analyses of our transcriptomics data uncovered important and previously unappreciated changes in the DC transcription factor landscape, thus revealing a novel *Leishmania* immune subversion strategy directly acting on transcriptional control of gene expression. Our data raise important questions on the dynamic and reciprocal interplay between *trans*-acting and epigenetic regulators in establishing permissive conditions for intracellular *Leishmania* infection and polarization of the immune response.

## Introduction

Dendritic cells (DCs) are essential components of the immune system initiating antigen-specific adaptive immune responses to foreign antigens and maintaining tolerance to self-antigens (1). They are recognized as key actors of the immune response to infection caused by viral, bacterial, and eukaryotic pathogens (2). Many of these infectious agents have evolved strategies to interfere with DC immune functions promoting their own survival. One strategy is represented by intracellular DC infection, which allows the pathogen to hide from immune recognition and to subvert DC signaling, gene expression, and immune activation. This is very well illustrated for the protozoan parasite *Leishmania amazonensis (L. am)* (3–5), one of the causative agents of diffuse cutaneous Leishmaniasis in South America (6, 7). Upon infection, DCs display reduced activation, maturation, *in vivo* and *in vitro* antigen-presenting capacities, and migration properties (8–11). These alterations were linked to the subversion of key signaling kinases, including STAT1/2/3 and ERK1/2 (10–14).

A number of pattern recognition receptors, including NOD-like receptors are involved in key steps of DC maturation and migration (15). NLRP3 (NOD-, LRR-, and pyrin domain-containing protein 3) is an intracellular sensor that is synthesized in response to a “priming signal” involving the engagement of cytokine or Toll-like receptors and further activated by pathogen- or damage-associated molecular patterns (PAMPs/DAMPs) such as ATP. This process triggers caspase-1 activation, which cleaves pro-IL-1β and pro-IL-18 into mature cytokines further secreted during the adaptive immune response (16, 17). While IL-1β favors efficient protective T cell responses (16, 18), notably Th17-mediated immunity (17, 19, 20), IL-18 potentiates IL-12-dependent development of IFN-γ-producing Th1 cells (17, 21). In DCs and macrophages, NLRP3 is activated in response to bacteria (22–24), fungi (25, 26), viruses (27, 28), and certain parasites (29). Recent studies evaluated NLRP3 activation in *Leishmania*-infected macrophages *in vitro* and *in vivo* (30, 31). While a previous study showed that *L. am* promastigotes caused NLRP3 activation in infected tissues *in vivo* (31), our recent study revealed that *L. am* amastigotes did not activate the inflammasome, neither *in vitro* in bone marrow-derived macrophages nor *in vivo* in lesional macrophages (30).

In contrast to macrophages, no information is available on the status of NLRP3 inflammasome activation and cell maturation in *L. am*-infected DCs, despite their essential roles in immune priming during *Leishmania* infection (32). Here we investigated these important open questions using primary, bone marrow-derived DCs (BMDCs) infected with *Ds*Red*2*-transgenic parasites that allowed for FACS-purification of infected cells (33). We thus overcame one of the major challenges in systems-level analysis of *Leishmania*-infected DCs represented by the low *in vitro* infection level (4, 12, 34, 35), which dilutes biological signals due to the presence of uninfected DCs (33). Transcriptomic analyses of sorted DCs using the Affymetrix GeneChip technology revealed that *L. am* infection causes important changes to the host cell transcription factor landscape that correlated with transcriptional activation of the alternative NF-κB pathway, but inhibition of the canonical NF-κB pathway as well as DC maturation and inflammasome activation.

## Materials and Methods

### Mice

Female BALB/c mice and Swiss *nu/nu* mice were purchased from Charles River (Saint Germain-sur-l'Arbresle, France). Female *Fcer1g* knockout (BALB/cByJMTac-*Fcer1g*tm1 N12) and corresponding wild type mice were purchased from Taconic (Taconic Biosciences, Inc.).

### Parasites, Bacteria, and Cell Lines

*L. amazonensis* strain LV79 (MPRO/BR/1972/M1841) genetically modified to stably express fluorescent *Ds*Red2 (33) were propagated in Swiss nu/nu mice. *L. amazonensis* amastigotes were isolated 2 months after infection from mouse footpad lesions purified as described (36). These amastigotes did not present antibodies at their surface (12). *Mycobacterium bovis* BCG (bacillus Calmette Guérin) was grown in Sauton medium, recovered as previously described (37) and stored at −80°C until use. The J558 cell line was cultured in Dulbecco's Modified Eagle's Medium (DMEM) supplemented with 10% heat-inactivated fetal calf serum (FCS; Dutscher, Brumath, France) to get GM-CSF rich supernatants.

### DC Culture and Infection by *L. amazonensis* Amastigotes

DCs were differentiated from bone marrow cells of 6-week-old wild type BALB/c or BALB/cByJMTac-*Fcer1g*tm1 N12 mice (BMDCs) (12). Briefly, bone marrow cells were seeded at 2 × 10^6^ cells per 100 mm diameter bacteriological grade Petri dish (Falcon, Becton Dickinson Labware, Franklin Lakes, NJ) in 10 ml of Iscove's modified Dulbecco's medium (IMDM; BioWhittaker Europe, Verviers, Belgium) supplemented with 10% heat-inactivated fetal calf serum (FCS; Dutscher, Brumath, France), 1.5% supernatant from the GM-CSF producing J558 cell line, 50 U/ml penicillin, 50 μg/ml streptomycin, 50 μM 2-mercaptoethanol, and 2 mM glutamine. Cultures were incubated at 37°C in a humidified atmosphere with 7% CO_2_. On day 6, suspended cells and loosely adherent cells were harvested using 1% EDTA (Versene) in PBS without Ca^2+^ and Mg^2+^ and cultured in the same plastic ware in complete IMDM supplemented with 10% of the primary culture supernatant. On day 10, cells were harvested following EDTA treatment as below and distributed in hydrophobic 6-well-plates (Greiner, St. Marcel, France) at a concentration of 9 × 10^5^ cells/ml in 3 ml of complete IMDM.

On day 14, freshly isolated *Ds*Red2-LV79 amastigotes or live BCG were incubated with BMDCs at ratios of 4:1 and 10:1, respectively. Amastigotes were opsonized or not with heat-inactivated immune serum prepared from *L. am*-infected BALB/c mice for 1 h at 4°C. Serum was removed by two washing steps in PBS (1,200 g, 10 min, 4°C). DC cultures were placed at 34°C for 24 h.

### Dendritic Cell Phenotyping by Flow Cytometry

BMDCs were resuspended in cold Dulbecco's PBS with 2% FCS and 0.05% sodium azide (PBS-FCS-Az) and transferred to a round-bottomed 96-well-plate (Costar, Corning, FR) at a concentration of 2–5 × 10^5^ cells/well. All subsequent steps were carried out on ice. Cells were incubated in PBS-FCS-Az supplemented with 10% donkey serum for 20 min. After centrifugation, DCs were incubated for 30 min in PBS-FCS-Az containing the primary biotinylated Abs: 2D7 (anti-CD11a/LFA-1 α-chain), M1/70 (anti-CD11b/CR3 α-chain), HL3 (anti-CD11c/p150, 95 α-chain), M1/69 (anti-CD24/HSA), 3/23 (anti-CD40), 3E2 (anti-CD54/ICAM-1), 16-10A1 (anti-CD80/B7-1), GL1 (anti-CD86/B7-2), 2G9 (anti-I-Ad/IEd) BD bioscience (San Diego, CA). Biotin-labeled IgGs, used as isotype controls, were obtained from BD bioscience and Caltag Laboratories (San Francisco, CA). After three washing steps, they were incubated with PBS-FCS-Az containing phycoerythrin-conjugated streptavidin for 30 min, cells were washed and treated with the CytoFix/CytoPerm reagent (BD bioscience) for 30 min. For dual staining including parasite detection, cells were also incubated either with the amastigote-specific 2A3-26 mAb (38) followed by fluorescein isothiocyanate-conjugated donkey anti-mouse Ig F(ab′)2 fragments or with the Alexa Fluor 488-conjugated 2A3-26 mAb to allow the detection of intracellular *Leishmania*. All washing and incubation steps were performed with Perm/Wash buffer (BD bioscience) supplemented with 10% donkey serum. Appropriate isotype controls (irrelevant rat, mouse, or hamster mAbs) were used at the same concentrations than those used for primary Abs. Flow cytometry results were acquired using a LSR Fortezza™ cytometer (Becton Dickinson, Mountain View, CA).

### Dendritic Cell Phenotyping by Fluorescence Microscopy

For epifluorescence and confocal microscopy analysis, DCs were collected by EDTA treatment, centrifuged, and resuspended in Dulbecco's PBS without Ca2^+^ and Mg2^+^. DCs were centrifuged onto poly-L-lysine-coated glass coverslips and incubated at 34°C for 30 min, before fixation with paraformaldehyde, permeabilization with saponin, and immunostaining (12). Cell preparations were mounted in Mowiol (Calbiochem, San Diego, CA) before analysis using an Axiophot Zeiss epifluorescence or a LSM 510 Zeiss confocal microscope. Confocal microscopy images were acquired and analyzed using the LSM 510 software (version 3.1).

### Preparation of DC Samples for High Speed Cell Sorting

After 5 min of contact with the Versen-EDTA solution at 34°C, DCs were carefully detached, resuspended at 4°C in Dulbecco's PBS with 2% FCS (PBS-FCS) and transferred to a 15 ml tube (Falcon; BD Biosciences, San Jose CA) at a concentration of 6 × 10^6^ cells/ml. Cells were centrifuged (300 g, 5 min, 4°C), and resuspended in PBS-FCS supplemented with 10% heat-inactivated donkey serum for 5 min. Cells were then incubated for 30 min in PBS-FCS containing 0.2 μg/ml of the anti-MHC class II monoclonal antibody (mAb) (M5/114) or the corresponding IgG2a isotype control mAb, both conjugated to PE-Cy5 (eBioscience). After two washes, cells were resuspended at 5 × 10^6^ cells/ml in PBS containing 3% FCS and 1% J558 supernatant. Cell aggregates were dissociated using a 70-μm filter (Falcon) and placed on ice until cell sorting.

### Cell Sorting

Once stained with the M5/114 mAb as described above, live DCs were sorted using a FACSAria (BD Biosciences) equipped with sealed sample injection and sort collection chambers that operate under negative pressure, and operated by the BD FACSDiva™ software (BD Biosciences). FSC and SSC parameters were displayed on a linear scale and used to discard cell debris. To avoid the sorting of cell doublets or cell aggregates, single cells were sequentially selected on SSC-H/SSC-W, and FSC-H/FSC-W dot plots. Infected DCs were sorted by selecting cells expressing surface MHC Class II molecules and containing *Ds*Red2 expressing intracellular amastigotes (576/26 bandpass filter). Uninfected DCs were sorted on the basis of MHC Class II expression using the same gating procedure as for infected DCs. Sorting conditions included: (i) sheath pressure of 70 Psi, (ii) flow rate of 7, and (iii) 70 μm nozzle tip. Cells were collected at 4°C in polypropylene tubes (BD Biosciences) previously coated with FCS (overnight incubation at 4°C). Sorted cells were immediately used for RNA isolation. All these experimental procedures were performed according to biosafety level two practices (BSL2) (39).

### RNA Integrity Quality Control

RNA isolation was performed with the RNeasy^+^ isolation kit (Qiagen) according to the manufacturer's instructions. Evaluation of RNA quality was carried out by optical density measurement using a Nanodrop device (Kisker, http://www.kisker-biotech.com) and by electrophoresis on a Lab-on-a-chip product using the Agilent 2100 Bioanalyzer (Agilent, http://www.chem.agilent.com). RNA Integrity Number (RIN) scores were monitored for each sample providing an objective and standardized measure of RNA quality on a scale of 1–10 (the value 10 corresponding to the highest quality) (40).

### Transcriptomic Analysis

RNA samples were subjected to GeneChip hybridization on the Mouse Genome 430_2.0 Array (Thermo Fisher Scientific) following the Affymetrix two-cycle labeling protocol. Affymetrix MIAME-compliant data have been made available through Gene Expression Omnibus databases (www.ncbi.nlm.nih.gov/projects/geo/, accession: GSE144039). Data processing, background correction, normalization, and signal quantification were carried out using RMA algorithm using the Bioconductor “affy” package version 1.62.0 (41). Differential expression was determined using R version 3.6.1 and the Bioconductor limma package version 3.40.6 (42). Raw *p*-values were adjusted for multiple testing using the Benjamini and Hochberg algorithm, and probesets with an adjusted *p*-value lower than 5% were considered differentially expressed.

### Gene Ontology Analysis

Raw probe-set identifiers were translated into ENTREZ prior to the enrichment analysis since gene-sets are defined by lists of ENTREZ gene identifiers. Additionally, ENTREZ genes linked to several probe-sets were associated with the probe-set having the most variable expression across replicates. Functional gene-set enrichment analysis was performed using the Fisher statistical test for the over-representation of differentially expressed genes (adjusted *P*-value lower than 5%). The genes lists were used to interrogate the gene-set collection of Gene Ontology (GO) annotations selected from the Molecular Signatures Database MSigDB v6.2 (43). Only gene-sets with an FDR lower than 0.05 were considered significantly enriched in differentially expressed genes.

### Network Analysis

Networks shown in Figure 7 were derived from the String Database (https://string-db.org) with text mining, neighborhood, experiments, gene fusion, databases, co-expression, and co-occurrence as active interaction sources. Network representation was performed with Cytoscape (V3.7.1). Network edges correspond to active interactions and are depicted as gray lines, whereas black arrows correspond to molecule interactions verified in the literature. The list of transcription factors, their co-regulators and epigenetic factors was created manually from different web sources (AnimalTFDB/OMICS_01856, Riken Transcription Factor Database, TRRUST v2, and mTFkb) (44–46) (Supplementary Table 1).

### Western Blotting

DCs were lysed in RIPA buffer (R0278, SIGMA) supplemented with a cocktail of anti-proteases and anti-phosphatases inhibitors (MS-SAFE, SIGMA). Proteins were resolved by SDS–PAGE on NuPAGE gels (4–12% Bis-Tris) in MOPS buffer and electroblotted onto polyvinylidene difluoride (PVDF) membranes in transfer buffer. Membranes were blocked with 5% fat-free milk in 1 × Tris-buffered saline containing 0.25% Tween 20 and then probed overnight at 4°C with the following primary antibodies: anti-NLRP3 (MAB7578, R&D Systems), anti-OPTINEURIN (polyclonal rabbit antiserum against amino-acids 84-164 of OPTN) (47), anti-IRAK1 (H-273, sc-7883, Santa Cruz), anti-MYD88 (ab2064, Abcam), anti-RELA (ab32536, Abcam) and anti-RELB (ab#1319 from Nancy Rice) (48), and anti-β-ACTIN (4970, Cell Signaling) antibodies.

Following incubation with the appropriate peroxydase conjugate secondary antibodies, membranes were revealed by SuperSignal West Pico reagent (ThermoFisher Scientific) in a high-resolution PXi machine (Syngene). Relative protein expression was calculated by densitometric analysis using the ImageJ software. The integrated density was measured on scanned gels with inverted images free of pixel saturation using a region of interest for each specific band. For every band, the ratio between the values obtained for the target protein and the β-actin normalization control was calculated and fold changes calculated using the control sample as calibrator, control values of uninfected and unstimulated samples being set to 1.

### Cytokine/Chemokine Profiling and Quantitation in Culture Supernatants

Cytokine/chemokine profiling was performed by the mouse XL cytokine array kit (R&D Systems) according to the manufacturer's instructions. Membranes were revealed by SuperSignal West Pico reagent (ThermoFisher Scientific) in a high-resolution PXi machine (Syngene). Semi quantitative analysis was performed with the Quickly & Easily Process Proteome Profiler™ Antibody Arrays software (R&D Systems) on the membrane scans. IL-1β quantification in the supernatants was performed by using the mouse instant ELISA kit (eBioscience) following the manufacturer's recommendations.

### Statistical Analyses

Two-sided Student's paired *t*-tests were used to compare data from flow cytometry experiments and gene expression studies performed on sorted samples (6 < *n* <13). A non-parametric Mann-Whitney bilateral *U*-test was used for gene expression comparisons on unsorted samples (*n* = 5).

## Results

### *L. amazonensis* Amastigotes Stall DC Maturation

**Figure 1:**
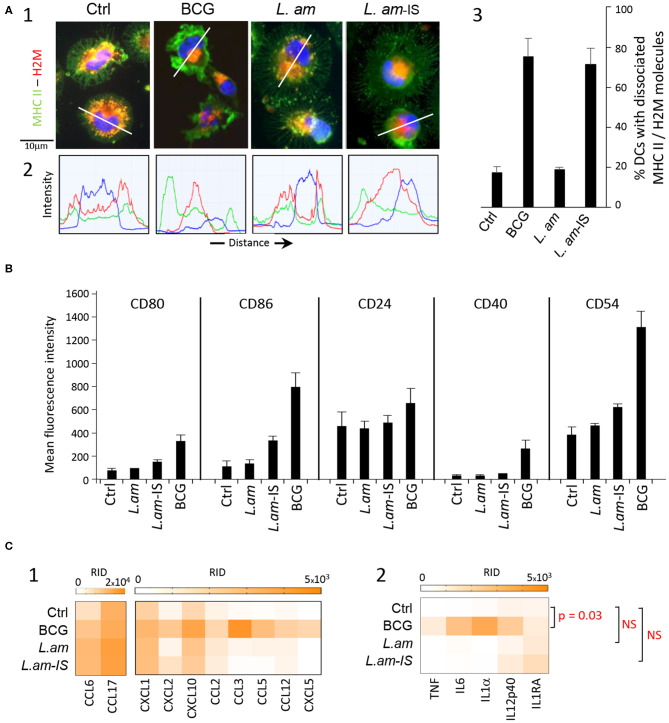
Multiparametric characterization of dendritic cell cultures. BALB/c-derived BMDCs alone (Ctrl) or incubated for 24 h with BCG or *L. amazonensis* amastigotes without (*L. am*) or with Ab-epsonization (*L. am*-IS) were analyzed. **(A)** Confocal microscopy imaging of DCs stained for MHC Class II and H2-M molecules. Images of representative DCs from the different conditions are displayed. (1) Merged images of the three detection channels showing MHC Class II molecules (green), H2-M molecules (red), and nuclear DNA (blue). (2) Intensity distribution of the three detected fluorescence signals along the transect line indicated in (1). (3) Percentage of DCs showing dissociated localization for MHC Class II and H-2M (*n* = 3 independent experiments). **(B)** Expression levels of co-stimulatory and adhesion molecules. Flow cytometric analysis was performed on DCs from the different culture conditions (*n* = 3–6 independent experiments). Mean (+ SD) fluorescence intensity (MFI) signals obtained for the corresponding stainings are shown. **(C)** Chemokine and cytokine determination in culture supernatants. The level of secretion was analyzed at 24 h post-infection in culture supernatants using the proteome profiler mouse XL cytokine array kit. Heat maps of background-corrected Raw Integrated Density (RID) values for technical duplicates are represented for chemokines (1) and cytokines (2). Scanned membranes of protein arrays are shown in Supplementary Figure 1. *P*-values for inter-group differences are indicated. NS, not significant.

We first assessed the maturation level of DCs in response to infection with lesion-derived *L. amazonensis* amastigotes (*L.am*). BMDCs were incubated with parasites without prior opsonization or were opsonized with immune serum obtained from *L. am*-infected BALB/c mice (*L.am*-IS), known to favor *Leishmania* uptake through Fcγ receptors and to facilitate the acquisition of protective immunity (12, 49). To evaluate the extent of DC maturation after 24 h of infection, we first stained DCs for MHC Class II molecules and the peptide-loading facilitator H2-M, which co-localize in immature but dissociate in mature DCs (50) (Figures 1A1,2, Supplementary Figure 1). Epifluorescence microscopy analyses revealed that these markers predominantly co-localize in discrete vesicles in the majority (80%) of DCs infected with non-opsonized amastigotes similar to uninfected control, but dissociate during infection with Ab-opsonized amastigotes (>70% of infected cells) as observed for BCG-treated, mature DCs (Figures 1A1–3).

We next quantified surface expression of costimulatory (CD80, CD86, and CD40) and adhesion (CD24 and CD54) molecules diagnostic for DC maturation using flow cytometry. As expected, BCG-infection strongly increased the surface expression of all markers compared to control (Figure 1B). Conversely, no significant increase in marker expression was observed in response to infection with non-opsonized *L. am*, thus confirming their stealthy entry (Figure 1B). Surprisingly, surface marker expression was only slightly increased in DCs infected with Ab-opsonized *L. am*, a feature however clearly dependent upon the presence of the Fcγ chain as no phenotypic cell surface modulation could be evidenced in Fcγ^−/−^ BMDCs infected with opsonized *L. am* parasites (Supplementary Figure 2). These slight changes observed at the DC surface were also contrasting with the clear maturation signal revealed by MHC Class II/H2-M dissociation (Figure 1A). This discrepancy indicates a stalled maturation process that was confirmed by the absence or lower secretion levels of a series of chemokines (CCL2, CCL3, CCL5, CCL12, LIX/CXCL5) and cytokines (TNF, IL6, IL1α, IL12p40, and IL1RA) in *Leishmania*-infected samples compared to BCG-infected DC cultures (Figures 1C1, 2, Supplementary Figure 3). These results further demonstrate that in contrast to BMDC infection *in vitro* with *L. braziliensis* promastigotes, *L. amazonensis* amastigotes do not induce TNF in non-infected bystander cells (51). Together these data reveal an important role of *Leishmania* opsonization in shaping the DC response, and uncover a novel parasite immune-subversion strategy to stall the DC maturation process.

**Figure 2:**
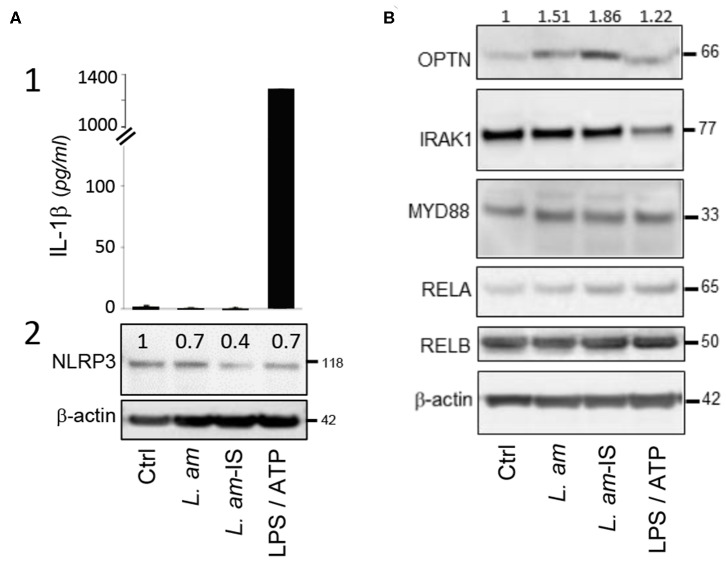
Analysis of protein abundance of key components of the TLR – NF-kB – NLRP3 axis. BMDCs alone (Ctrl), treated with LPS/ATP for inflammasome activation, or incubated for 24 h with either BCG or *L. amazonensis* amastigotes without (*L. am*) or with Ab-opsonized (*L. am*-IS) were analyzed. **(A)** Analysis of DC inflammasome priming and activation. The status of inflammasome priming and activation was analyzed following IL-1β secretion into culture supernatants and quantifying NLRP3 expression in cell lysates. (1) IL-1β was quantified by ELISA and data displayed as the mean quantity of IL-1β ± SEM (technical duplicates of one representative experiment, *n* = 2 independent experiments). (2) NLRP3 detection by Western Blotting. Values shown on top of the lanes indicate the relative abundance of the normalized value of NLRP3 to β-actin compared to control DCs (Ctrl). **(B)** Protein expression of key members of the TLR-NF-κB signaling pathway. The abundance of OPTN, IRAK1, MYD88, RELA, and RELB in cell lysates was analyzed by Western Blotting. Values shown on top of the lanes indicate the relative abundance of the normalized value of OPTN protein to β-actin compared to control DCs (Ctrl) for a representative Western Blot (*n* = 2 independent experiments).

### *L. amazonensis* Avoids Inflammasome Activation in DCs

The NOD-like receptor protein NLRP3 is a cytosolic sensor that triggers maturation and secretion of pro-inflammatory IL-1β (52). Contrary to the LPS/ATP-treated positive control, infection with Ab-opsonized or non-opsonized parasites does not result in IL-1β secretion (Figure 2A1), demonstrating the absence of inflammasome activation as we observed previously in macrophages (30). DCs infected with Ab-opsonized amastigotes even showed a significant reduction of NLRP3 expression (Figure 2A2), which correlated with increased expression of Optineurin (OPTN), an inhibitor of the TLR-induced, canonical NF-κB pathway. In contrast, infection neither affected expression of key activators of the TLR pathway (IRAK1, MYD88, and RELA) nor RELB, a key element pivotal for DC differentiation, maturation, and MHC Class I-restricted presentation (53, 54) (Figure 2B). Our data thus identify induction of OPTN expression as a potential key mechanism of *Leishmania* to escape detection by the DC TLR–NF-κB–NLRP3 innate immune axis.

### Transcriptome Profiling of *L. amazonensis*-Infected DCs

**Figure 3:**
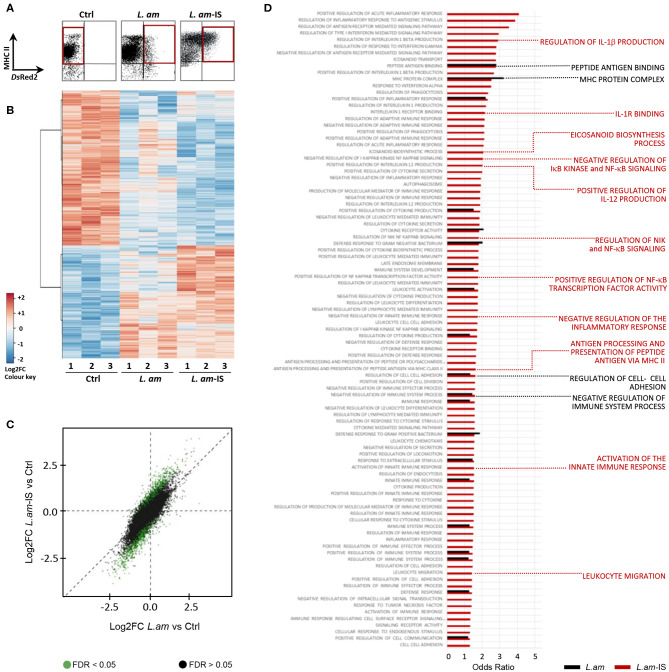
Gene expression analysis in infected DCs enriched by high speed cell sorting. BMDCs alone (Ctrl) or incubated with BCG or *Ds*Red2-transgenic *L. amazonensis* amastigotes without (*L. am*) or with Ab-opsonized (*L. am*-IS) were analyzed. After 24 h, live cells were carefully detached, stained with anti-MHC Class II mAb and sorted with a FACSAria under a BSL-2 cabinet. Sorted DCs were lyzed and total RNA was extracted for transcriptomic analysis. Three independent biological experiments were performed. **(A)** DC isolation by Fluorescence Activated Cell Sorting. The region of interest (red gates) for the sorting of non-infected and infected DCs was defined by the expression of MHC II as determined in uninfected DCs (control, Ctrl), and the presence of the *Ds*Red2 signal of intracellular parasites. **(B)** Global overview of gene expression in sorted samples. Heatmap visualizing differentially expressed Affymetrix probe sets (5% threshold) for sorted triplicate samples (indicated by the number). The color code corresponds to the values of the row-centered expression matrix. **(C)** Illustration of the expression changes between control, *L.am* and *L.am*-IS infected DCs. Log2FC of *L.am*-IS vs. control are plotted (Y-axis) against the log2FC of *L.am* vs. control (X-axis), and dots are displayed in green for probesets differentially expressed (adjusted *p* < 0.05) between *L.am*-IS and *L.am*. The dispersion of green dots indicates a more pronounced transcriptional modulation in DCs infected by Ab-opsonized amastigotes. **(D)** GO enrichment analysis related to DC immune processes in response to *Leishmania* infection. Odds Ratios for key DC biological processes enriched in *L.am* and *L.am*-IS infected DCs compared to control are displayed as black and red bars, respectively. Key DC processes are indicated by the red and black labels.

We next performed transcriptomic analyses on *Leishmania* infected DCs to gain further insight into the mechanisms underlying their stalled maturation. A major challenge in the systems-level analysis of DC/*Leishmania* interaction is to avoid the dilution of any infection-related signal in the analysis of heterogeneous DC populations with low percentage of *Leishmania*-hosting DCs (33). Indeed, we observed an infection efficiency of 10.8 ± 1.8 and 19.5 ± 2.5 % using non-opsonized and Ab-opsonized amastigotes, respectively. To overcome this challenge, we used *Ds*Red2 transgenic parasites to purify infected DCs by high-speed cell sorting as previously designed and validated (33). This approach was based on a bi-parametric analysis and sorting, using MHC Class II expression as a common marker for sorting control and infected DCs, and *Ds*Red2-fluorescence as a marker for intracellular infection (MHC Class II positive, *Ds*Red2 positive DCs, see Figure 3A, *L. am* and *L. am*-IS conditions). Total RNA was isolated from purified DCs in three independent, biological experiments, tested for quality (Supplementary Figure 4A) and processed for probe-set hybridization and quantification by the Affymetrix microarray technology.

Cluster analysis of modulated probe-sets revealed highly reproducible changes in the DC transcript profiles in response to non-opsonized and opsonized amastigotes, with respectively 2,077 and 3,293 genes showing differential expression compared to uninfected samples (*p* < 0.05, |FC| > 1.5, Figure 3B, Supplementary Figure 4B). 74.1 and 70.95% of these regulated genes were down modulated in DCs infected by non-opsonized and Ab-opsonized amastigotes, respectively. Opsonization generally enhanced the expression changes observed in DCs infected with non-opsonized parasites (Figure 3C, green dots, and Supplementary Figure 4B). Finally, gene set enrichment analysis revealed that non-opsonized and Ab-opsonized amastigotes interfered with numerous DC processes including those implicated in the regulation of the MHC Class II protein complex, peptide antigen binding, and cell adhesion (Figure 3D and Supplementary Table 2). Interestingly, as judged by the decrease in transcript abundance, DC infection with Ab-opsonized amastigotes interfered with various immune-related processes, including NF-κB signaling and the production of IL-1β, IL-12, and eicosanoids.

### *L. amazonensis* Infection Affects Genes Linked To DC Maturation and Pro-inflammatory Response

**Figure 4:**
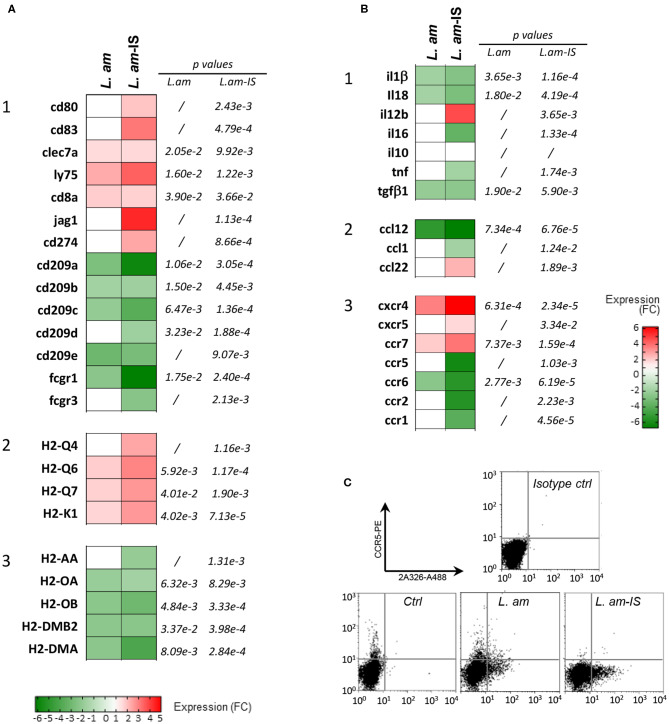
Expression analysis of genes related to the maturation process in *Leishmania*-infected DCs. **(A,B)** Affymetrix analyses were performed on sorted DCs infected with non-opsonized (*L. am*) or Ab-opsonized (*L. am*-IS) *Ds*Red2-transgenic amastigotes. Transcriptional modulation of genes involved in the DC maturation process are displayed as the mean fold change values calculated using uninfected sorted DCs as a calibrator. **(A)** Expression of genes related to maturation and antigen presentation. Heatmaps representing the expression modulation of genes coding for costimulatory molecules and surface receptors (1), MHC I (2), and MHC II (3) molecules. **(B)** Expression of chemokine and cytokine genes. Heatmaps representing the modulation of genes coding for cytokines (1), chemokines (2), and chemokine receptors (3). **(C)** FACS analysis of CCR-5 expression levels. DCs from unsorted cultures were analyzed by FACS for the expression of CCR5, a marker that is rapidly lost during the maturation process. Infected cells were detected using the amastigote-specific, Alexa Fluor-488 conjugated mAb 2A3-26.

The fold changes observed in RNA abundance between infected and control DCs were calculated and visualized for genes coding for co-stimulatory molecules, key DC markers, Fcγ receptors and MHC molecules (Figure 4A) as well as cytokines, chemokines and their receptors (Figure 4B). Overall, expression changes observed during amastigote infection were generally enhanced by opsonization, with a mean |linear FC| = 2.116 ± 0.1004 and 3.138 ± 0.3244 for *L.am* and *L.am*-IS, respectively (*p* > 0.0013) as illustrated for two *Leishmania* receptors involved in parasite uptake, the C-type lectin CD209 and the Fcγ receptor 1 (55, 56) (Figure 4A1). In contrast, transcripts coding for the costimulatory molecule *cd80* and the immune modulator *cd83* (57) were exclusively increased in response to Ab-opsonized parasites, confirming initiation of the DCs maturation process in accordance to data shown in Figure 1.

Amastigote infection increased the abundance of host cell transcripts for molecules involved in classical MHC Class I-restricted antigen presentation, including MHC Class I alpha chain molecules (H2-Q4, Q6, Q7, and H2-K1), and LY75 known to favor antigen cross-presentation (58) (Figures 4A1, 2). Transcripts for CD8α, a marker expressed on cross-presenting DC subsets (59) was also increased. In contrast, reduced transcript abundance was observed for MHC class II alpha chains and H2-M molecules (H2-DMB2 and H2-DMA, Figure 4A3) known to load peptides onto conventional class II molecules (60).

The changes observed in the cytokine/chemokine transcript profile further supported the stalled DC maturation phenotype observed in Figure 1: Increased abundance of *il12b* transcripts correlated with the slight increased secretion of IL12p40 (Figures 4B1, 1C2). In contrast, transcripts for other cytokines known to be secreted during DC maturation (*tnf, il6*) (61, 62) and after inflammasome activation (*il1*β*, il18*) were down modulated during infection, in accordance to the absence of secretion of these cytokines.

Modulation of some chemokine transcripts, such as *ccl12* or *ccl22* was observed (Figure 4B2), which did not translate into a corresponding increase in secreted proteins after only 24 h of infection (Supplementary Figure 3). A number of transcripts for chemokine receptors responded to *Leishmania* infection (Figure 4B3), including receptors that are crucial for DC migration: (i) increased expression was observed for *ccr7, cxcr4*, and *ccr2*, with the latter one previously linked to differentiation of protective DCs during *L. braziliensis* infection (63), and (ii) decreased expression was observed for *ccr5* and *ccr6*, which was confirmed at the protein level for CCR5 by FACS analysis (Figure 4C). Amastigotes (notably upon Ab-opsonization) thus seem to promote DC motility, which could favor parasite dissemination and visceralization (64).

### *L. amazonensis* Establishes an Anti-inflammatory Phenotype in Infected BMDCs

**Figure 5:**
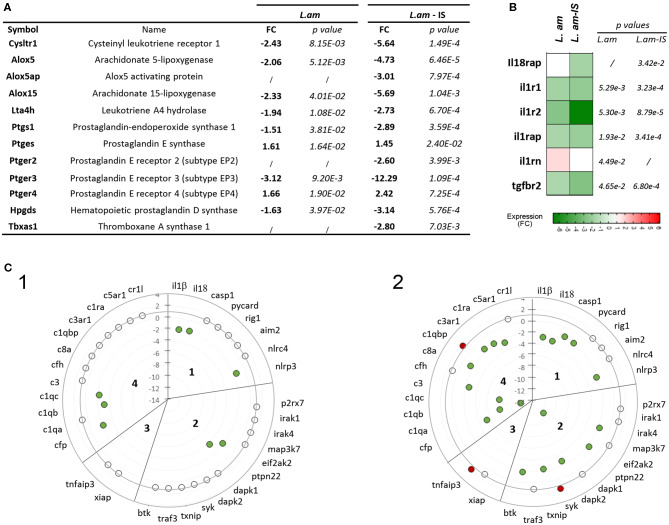
Induction of an anti-inflammatory expression profile in *L*. am-infected BMDCs. Transcriptional modulation observed in the microarray analysis are displayed as mean fold change values calculated using uninfected sorted DCs as calibrator. **(A)** List of differentially expressed genes associated to eicosanoid production. Linear fold changes and *p*-values are indicated for *L. am* and *L. am*-IS sorted DCs. **(B)** Heatmap of transcriptional modulations observed for cytokine receptor genes. **(C)** Transcriptional profiling of genes related to NLRP3 inflammasome priming and activation pathways. Radar plots displaying the modulation of gene expression in sorted DCs infected with non-opsonized (*L. am*, panel 1) and Ab-opsonized (*L. am*-IS, panel 2) *Ds*Red2-transgenic *L. am* amastigotes. The sub-sections correspond to inflammasome-related cytokines and inflammasome components (section 1), NLRP3/ASC activators (section 2), NLRP3 inhibitors (section 3), and components of the complement system (section 4). Unmodulated transcripts are indicated by open dots, whereas up-modulated and down-modulated transcripts are indicated by red and green dots, respectively.

Transcript profiling revealed that *L. am* infection triggered an anti-inflammatory expression pattern in DCs irrespective of their opsonization status. First, many transcripts related to eicosanoid production were down modulated (Figure 5A), including those leading to the synthesis of the leukotriene LTB4, known to amplify NF-κB-mediated responses in macrophages (65) and to be involved in the control of *L. am* infection (66). The increase in *ptges* (Prostaglandin E Synthase) indicates a potential increase in PGE2 levels, which is involved in the suppression of the inflammatory response during human *Leishmania* infection (67). Second, an anti-inflammatory pattern is further supported by (i) down modulation of cytokine receptors involved in signaling cascades leading to NF-κB activation (IL-1 and IL-18 receptor associated proteins and IL-1 receptors), and (ii) up modulation of the IL-1 receptor antagonist (*il1rn*), known to bind to IL-1 receptors and prevent downstream signaling (68) (Figure 5B). However, no quantitative difference could be demonstrated for secreted IL1RA as assessed by Cytokine Array (Supplementary Figure 3). Third, RNA abundance for the immune-regulatory molecule CD200-known to dampen host microbicidal responses (69) - was increased in DCs infected by Ab-opsonized amastigotes (linear FC = 4.02; *p* = 8.80E-4). Finally, our data revealed for the first time a coordinated transcriptomic subversion of the inflammasome in DCs infected with non-opsonized (Figure 5C1) or Ab-opsonized parasites (Figure 5C2), as judged by down modulation of transcripts of various inflammasome components and related cytokines (*caspase-1, nlrp3, il1*β, *il18*, quadrants 1 in panels C1 and C2), of NLRP3/ASC activators (quadrant 2), and of various complement components involved in inflammasome activation and IL-1β secretion (70, 71) (quadrant 4). In contrast, increased transcript abundance was observed for the NLRP3 inhibitor *tnfaip3* (panel C2, quadrant 3) and *c1qbp*, an inhibitor of the classical complement pathway (panel C2, quadrant 4), thus further reinforcing the anti-inflammatory phenotype. In agreement with the absence of IL-1β secretion (Figure 2), this profile indicates that *L. am* infection suppresses NLRP3 priming and activation at four different levels, reminiscent to our recent observation in primary macrophages *in vitro* and *in vivo* (30). We next assessed the transcriptional effect of infection on the TLR-NF-κB signaling pathway given its essential role of NLRP3 priming (72, 73).

### *L. amazonensis* Amastigotes Subvert NF-κB Mediated Signaling at The Transcript Level

**Figure 6:**
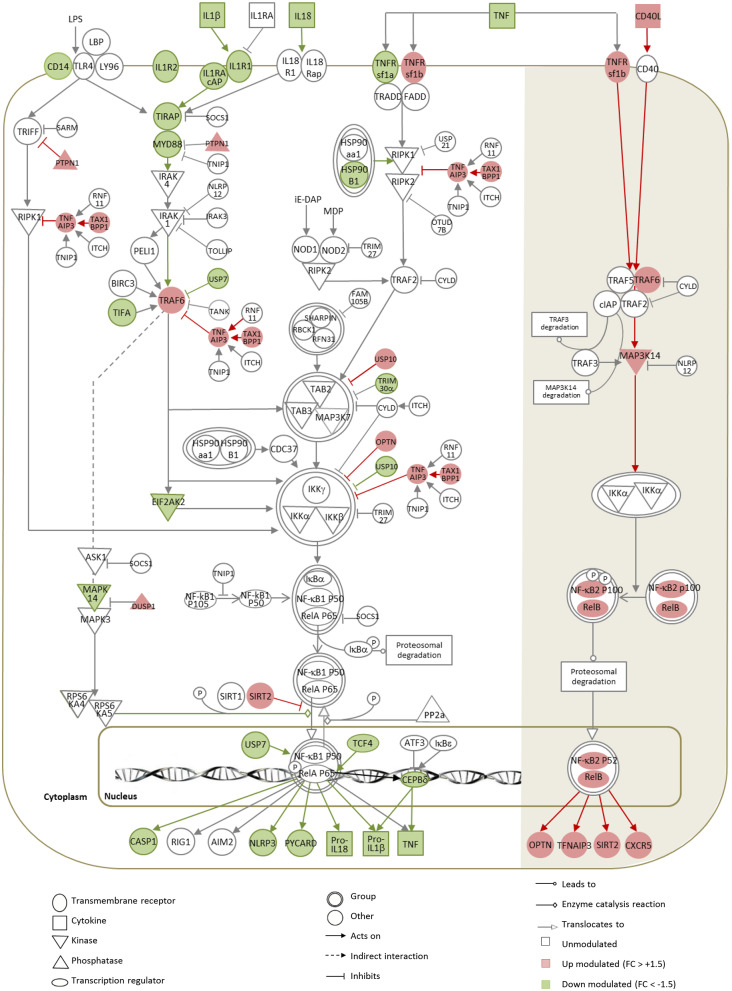
Gene expression map of the NF-κB pathway in DCs infected with Ab-opsonized amastigotes. Significant modulations calculated between *L.am*-IS-infected and uninfected BMDCs are represented by the color code, with red indicating up-regulated (linear FC > +1.5) and green down-regulated (linear FC < -1.5) genes. Symbols, lines, and color codes are defined in the legend. White (left) and shaded (right) areas correspond to the classical and to the alternative NF-κB pathways, respectively.

We investigated the impact of infection and parasite opsonization on the TLR/NLRP3-activated, canonical NF-κB pathway, and on the CD40/TNFRSF1b-activated, alternative NF-κB pathway linked to cross-presentation (74, 75). A pleiotropic subversion of the canonical NF-κB pathway was observed following infection with both Ab-opsonized and non-opsonized amastigotes (Figure 6, Supplementary Figure 5, respectively). Similarly to observations in *Leishmania*-infected macrophages (30), the TLR-NF-κB-NLRP3 axis was inhibited in a dual fashion: (i) by downregulating genes encoding for activators (IL-1 receptors, TIRAP, MYD88, TIFA, EIF2AK2, USP7) and (ii) upregulating genes encoding for negative regulators of the NF-κB pathway (OPTN, TNFAIP3, TAX1BP1, PTPN1, USP10) (Figure 6), which was validated at protein level for OPTN (Figure 2B), a key inhibitor of NF-κB-mediated immune signaling (76), but not for MYD88 (Figure 2B).

In contrast, the observed transcript profile indicates activation of the alternative NF-κB pathway at all levels of the signaling cascade, from the TNFRsf1b surface receptor to the NF-κB2 and RelB nuclear factors (Figure 6). The corresponding transcripts were significantly up modulated in response to infection, but no quantitative changes could be evidenced by WB for RELB (Figure 2B), whereas the main negative regulator of this pathway *nlrp12* (77) was not affected. The activation of the alternative NF-κB pathway likely causes increased transcription of OPTN, TNFAIP3, and SIRT2, which all are known to further counter-act the activity of the classical NF-κB pathway.

### Involvement of Transcription Factor Regulation in Dendritic Cell Subversion by *Leishmania*

**Figure 7:**
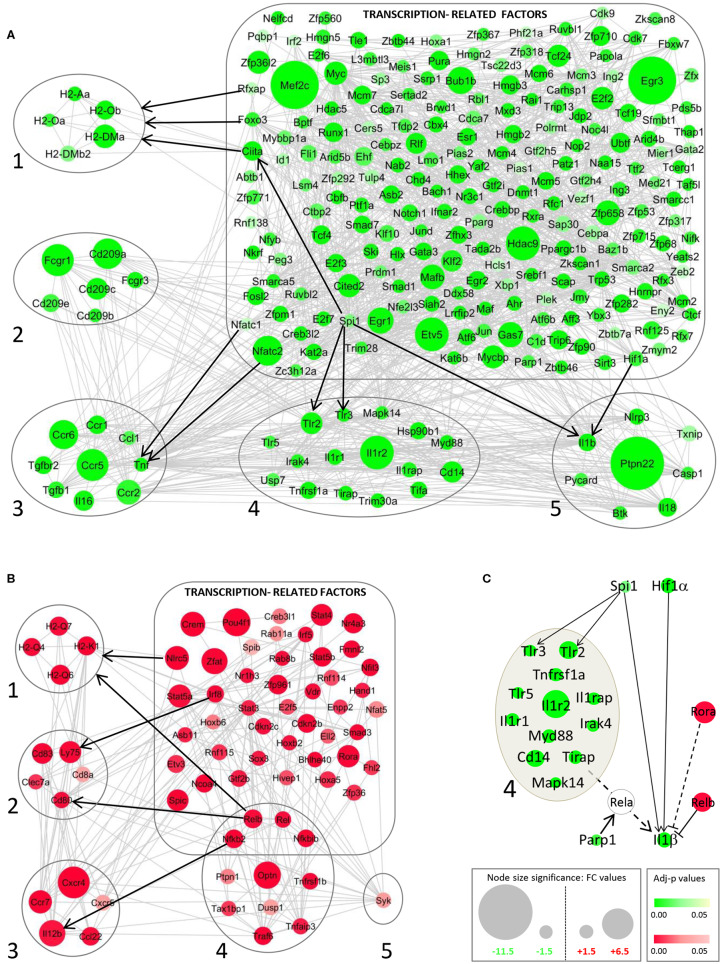
STRING network analysis for modulated genes of transcription-related factors (TRFs) in DCs infected with Ab-opsonized amastigotes. Networks for down-modulated (green) **(A)** and up-modulated genes (red) **(B)** are shown. Interactions between TRFs and MHC II (1), DC markers/receptors (2), cytokine/chemokine receptors (3), TLR/Cytokine receptors and the NF-κB pathway (4), and inflammasome-related molecules (5) are depicted. Gray lines correspond to active interactions (settings used for STRING analysis) and black arrows exemplify key TRF – target gene interactions validated by publication. **(C)** Integrative network of modulated TRFs and their related target genes involved in regulation of the IL-1β gene. Dotted lines correspond to indirect interactions (mediated by RelA, related to the TLR/NF-κB axis, group 4) on the IL-1β gene.

The important changes in DC gene expression during *L. am* infection primed us to investigate the potential underlying mechanism of this profound transcriptomic reprogramming. We therefore mined our data sets for changes in the expression profile of Transcription-Related Factors (TRFs), including Transcription Factors (TFs), transcriptional co-regulators and epigenetic factors. Among 2,243 TRFs analyzed (Supplementary Table 1), 11.3 and 15.5% showed significant changes in transcript abundance in DCs infected with non-opsonized and Ab-opsonized amastigotes, respectively. These changes correlated with the expression levels of their target genes as revealed by network analysis (Figure 7, Supplementary Figure 6). For example, decreased expression of the TNF gene correlates with decreased expression of the NFATC1 and NFATC2 transcription factors that regulate key DC immune functions (Figures 7A1–5) (78). Likewise, coordinated down-modulation of the MHC Cl II genes H2-Aa, H2-Oa, H2-Ob, H2-DMb2, and H2-DMa (Figure 7A) correlates with reduced expression of (i) the Class II Major Histocompatibility Complex Transactivator (CIITA) essential for transcriptional activity of the MHC class II promoter (79), (ii) the Forkhead transcription factor FOXO3a that is a key component of the MHC II enhanceosome (80), and (iii) regulatory factor X-associated protein (RFXAP) that binds to the X-box of MHC II promoters (79). These simple regulatory relationships are likely part of more complex regulatory cascades and networks, as suggested by the down-regulation of CIITA itself that could result from the reduced expression of the ETS-domain transcription factor SPI-1 (81) (Figure 7A). Vice versa, TF upregulation was correlated with increased target gene expression, as exemplified by the regulatory relationship between NLRC5 and MHC Class I molecules (82, 83), REL and IL12b (84), RELB and CD80 and MHC Class I molecules (85) or IRF8 and LY75 (86) (Figure 7B).

Our network analysis further reveals that reduced IL-1β expression is likely the result of a dichotomic regulatory event, with *L. am* infection reducing the expression of its direct transcription factors (SPI-1 and HIF-1α) (87, 88), while at the same time increasing the expression of the TFs RELB and RORA that have a negative effect on IL-1β expression (89–91) (Figure 7C). Unlike previously observed in *L. am*-infected macrophages (30), the expression of the pro-inflammatory NF-κB family member RELA is not modulated in BMDCs. However, the reduced expression of (i) upstream actors of the classical NF-κB pathway, including TLR2 and TLR3 that are also expressed under the control of SPI-1 (92, 93), and (ii) the Poly(ADP-ribose) polymerase-1 (PARP1) gene, which encodes for a nuclear chromatin-associated protein known to co-activate NF-κB-dependent transcription (94), likely prevents RELA-dependent IL1β gene expression (Figure 7C).

Together, these data reveal that the subversion of the DC transcriptomic landscape during *Leishmania* infection may be the consequence of subversion of key TFs that regulate the host cell immune response.

## Discussion

**Figure 8:**
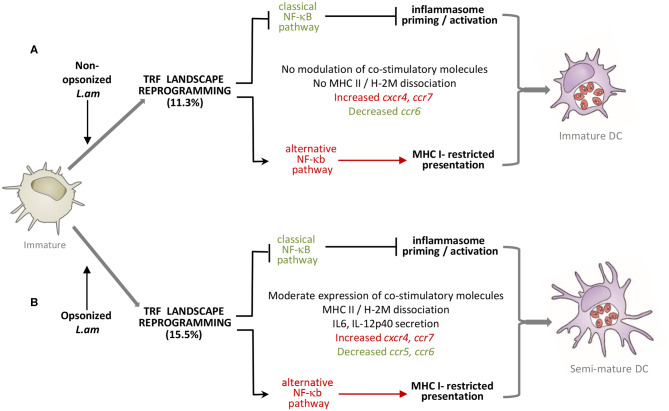
Model of DC subversion by *L. am* amastigotes. Transcriptomic and phenotypic modulations induced by non-opsonized **(A)** and Ab-opsonized **(B)** *L. am* amastigotes in immature BMDCs are shown. In both cases, inhibition of the classical NF-κB pathway is linked to prevention of inflammasome priming and activation. The alternative NF-κB pathway is favored in both infection conditions, likely promoting MHC I-restricted antigen presentation.

Using a FACS-based sorting procedure and applying systems analyses on transcriptomics data, we uncovered a novel mechanism of DC immune-subversion by non-opsonized and Ab-opsonized, virulent *Leishmania amazonensis* amastigotes. This subversion targets transcription-related factors (TRFs), which in turn causes important changes in expression of immune-related genes, interferes with DC maturation (Figure 8) and favors persistent parasite infection.

*Leishmania* parasites are capable to alter key immune functions of DCs, including maturation and migration properties (8–11). The subversion of these essential functions are mediated by the inhibition of various signaling pathways, including pro-inflammatory transcription factors of the STAT and NF-κB protein families (10, 11, 13, 14, 95, 96). Our data largely extend this list to many other TFs, i.e., *spi1, tcf4, myc, zbtb46, irf2, irf8, relb, pparg, spib*, or *nfil3* (97) that show parasite-driven inhibition of expression in infected DCs, raising important questions on the underlying mechanisms that allow such coordinated modulation of the TRF landscape. In macrophages, *Leishmania* infection has profound effects on the macrophage epigenetic profile, both at the level of DNA methylation (98) and H3 histone post-translational modifications (30, 99, 100). We recently provided first evidence that *L. am* amastigotes induce histone H3K9/14 hypo-acetylation and H3K4 hypo-trimethylation at promoters of NF-κB-related, pro-inflammatory genes in infected macrophages *in vitro* and in infected tissues *in vivo* (30). Based on these results, it is interesting to speculate that the expression changes in TRFs are caused by changes in the activity of histone modifying enzymes (HMEs) or DNA methyltransferases (DNMTs). Indeed, epigenetic regulation controls DC development, immune functions and phenotypic heterogeneity (101), and *L. am* may have evolved strategies to exploit this remarkable plasticity of DCs by interfering with the host cell's epigenetic profile. Our data provide first insight into a possible, reciprocal regulatory relationship between TRFs and HME/DNMT activities that may govern DC reprogramming: Mining our data sets we observed reduced expression in infected DCs of *dnmt1*, the histone deacetylases *hdac5* and *hdac9, sirt3*, and various TFs known to remodel the epigenetic landscape such as *cebpa* or *nfatc2* (102, 103).

The interplay between transcriptional and epigenetic regulation in establishing an anti-inflammatory phenotype in *L. am*-infected DCs is further illustrated by the factors controlling IL-1β expression, notably the NF-κB family members *rela* and *relb*. While the activation of the canonical NF-κB pathway depends on the rapid and transient nuclear translocation of RelA/p50 dimers, the non-canonical pathway is activated in a slow and persistent manner via a RelB/p52 complex (77, 104). This second pathway plays a critical role in regulating immune homeostasis, and its dysregulation contributes to inflammatory and autoimmune diseases (77, 105–107) suggesting that RelB may act as a repressor of NF-κB-responsive gene expression. Indeed, our data link increased expression of *relb* with inhibition of IL-1β expression during *Leishmania* infection, likely by changing the chromatin structure at the IL-1β promoter causing epigenetic silencing (89, 90).

Subversions of the TRF landscape in *L. am-*infected DCs had a profound effect on the host cell immune status, in particular on the TLR/NF-κB immune axis - a key signaling pathway regulating DC functions (16, 108, 109). This pathway was inhibited both at the transcriptional and signaling levels, resulting in reduced expression and secretion of chemokines and pro-inflammatory cytokines, absence of inflammasome activation, and stalling of the maturation process in *L. am*-infected DCs (Figure 8). Increased expression of TNFAIP3 and OPTN, two negative regulators of the TLR/NF-κB/NLRP3 axis, indicates that this pathway may be even suppressed in infected DCs as previously demonstrated in *L. am*-infected macrophages (30). Such a suppression is further sustained by the down-modulation of positive regulators of the TLR-classical NF-κB axis (e.g., IL1 receptors, TNFRsf1a, TIRAP, MYD88, TIFA, USP7) and increased expression of members of the alternative NF-κB pathway (e.g., TNFRsf1b, TRAF6, MAP3K14, NF-κB2, RelB), a similar regulatory dichotomy we recently uncovered in *L. am*-infected macrophages (30). Surprisingly, the anti-inflammatory state observed during DC infection was further enhanced by parasite Ab-opsonization, a *Leishmania*-specific signature that contrasts with the efficient DC maturation and inflammasome activation observed with other intracellular pathogens, including *Cryptococcus neoformans, Staphylococcus aureus, Escherichia coli* or *Francisella tularensis*, whose opsonization triggers IL-1β secretion (110–112). In contrast, MHC Class I-restricted presentation and cross- presentation seems to be promoted in *L. am*-infected DCs, confirming previous reports (113, 114). Based on our data, this response may be favored by increased expression of the mannose receptor LY75 (58), members of the alternative NF-κB pathway (115, 116), MHC Class I molecules, NFIL3 (117), the small GTPase RAB11A involved in receptor signaling (118), and the decreased expression of the m^6^A-marked mRNA binding molecule YTHDF1 (119, 120).

In conclusion, we describe a new *Leishmania* immune subversion strategy resulting in stalled DC maturation and pleiotropic inhibition of the TLR/NF-κB/NLRP3 axis, which may have important phenotypic and immunologic consequences: Preventing IL-1β secretion may hamper expansion, survival, and migration of antigen-primed CD4^+^ and CD8^+^ T cells, and Th1, Th2, and Th17 differentiation (18, 20, 121). Indeed, we observed increased transcript expression of *cd8a, ly75* (DEC205), *jag1*, and *cd274* in response to *L. am* infection, suggesting the induction of tolerogenic DCs (122, 123), which may favor the differentiation of anergic or regulatory T cells (124, 125), thus causing immune suppression and favoring *Leishmania* infection and immunopathology (126). Our results will incite future studies aimed to characterize the transcriptional landscape and antigen presenting capacity of DCs *in vivo* that can likely be modulated directly by intracellular *Leishmania*, indirectly by the uptake of parasite remnants, or by the local immune response, notably pro- (e.g., TNF) and anti- (e.g., IL-10) inflammatory factors produced by bystander cells. Our study will open interesting new avenues for the design of anti-parasitic immuno-therapies targeting DC epigenetic and transcriptional control to rescue the DC's key functions in mounting an efficient, anti-leishmanial T cell response.

## Data Availability Statement

The datasets generated for this study can be found in the GEO: GSE144039; secure token= khahiiccdjgjxyx.

## Ethics Statement

All animals were housed in A3 animal facilities according to the guidelines of Institut Pasteur and the “Comité d'Ethique pour l'Expérimentation Animale” (CEEA) and protocols were approved by the “Ministère de l'Enseignement Supérieur; Direction Générale pour la Recherche et l'Innovation” under number 2013-0047 and by the Animal Care and Use Committee at Institut Pasteur of Shanghai Animal Care.

## Author Contributions

HL, EP, GMi, GS, and GMe: study design. TR, HL, and P-HC: acquisition of data. HL, EP, TR, KK, and AL: analysis and interpretation of data. HV, EP, and HL: statistical analyses. HV and EP: organized the database. GMi and RW: material support. HL, EP, TR, KK, P-HC, and J-YC: technical support. HL, EP, GMi, GMe, and GS: drafting of the manuscript. All authors contributed to manuscript revision, read and approved the submitted version.

## Conflict of Interest

The authors declare that the research was conducted in the absence of any commercial or financial relationships that could be construed as a potential conflict of interest.

## Supplementary Material

Supplementary Table 1 List of transcription factors and co-regulators. Gene symbols are listed with their Ensembl reference and short description.

Supplementary Table 2 Partial list of GO gene sets highlighted in *Leishmania*-infected DCs. Gene-level results corresponding to 18 selected GO terms significantly (*p* < 0.05) evidenced in DCs infected with *Leishmania amazonensis* amastigotes (*L. am* and *L. am*-IS) are presented. The summary sheet describes the GO terms and their corresponding sheet (S2 to S19). For each GO term, genes are listed with their Entrez number, probe-set ID, Fold Changes (Log base 2) and adjusted *p* values for both *L. am* vs Ctrl and *L. am*-IS vs Ctrl comparisons. Color code indicates *p* values below the 5% threshold level.

Supplementary Figure 1 Evaluation of the maturation status of DC cultures by epifluorescence microscopy. BALB/c-derived BMDCs alone (Ctrl) or incubated for 24 h with BCG (BCG) or *L. amazonensis* amastigotes without (*L. am*) or with Ab-opsonized (*L. am*-IS) were subjected to epifluorescence microscopy imaging of MHC Class II and H2-M molecule staining. Representative DCs from the different conditions are displayed as merged images of the 3 detection channels showing MHC II molecules (green), H2-M molecules (red), and nuclear DNA (blue).

Supplementary Figure 2 Influence of the γ chain subunit of FcgammaRI, FcgammaRIII, and Fcepsilon RI receptors on the phenotype of DC cultures. **(A)** Flow cytometric comparison of surface marker expression of BMDCs from wild type mice from Charles River (CR) and Taconic (Tac) laboratories. Histograms show the mean fluorescence intensity of the indicated markers. Numbers represent the percentage of DCs expressing the corresponding marker. Note the similarity of the profiles between both sources of BALB/c mice. **(B)** Phenotypic analysis of DCs derived from wild type (gray histograms) and Fcγ^−/−^ (black histograms) mice from Taconic laboratories. DCs alone (Ctrl) or incubated for 24 h with BCG or *L. amazonensis* amastigotes without (*L. am*) or with Ab-opsonization (*L. am*-IS) are shown. Histograms correspond to the mean fluorescence intensity of the indicated markers. Numbers represent the percentage of DCs expressing the corresponding marker. Note that the absence of the γ chain abrogates the marker increase observed in *L. am*-IS-infected DCs from WT background.

Supplementary Figure 3 Results of cytokine array analysis of DC culture supernatants. Uninfected BMDCs (ctrl, **A**), or BMDCs infected with non-opsonized *L. am* amastigotes **(B)**, Ab-opsonized *L. am* amastigotes **(C)** or live BCG **(D)** are shown. Supernatants were analyzed using the mouse XL cytokine array kit. Pictures of the membranes are shown after revelation and scanning. Cytokines and chemokines already expressed in control cultures are labeled in black. Those specifically detected in the BCG-treated culture are labeled in red.

Supplementary Figure 4 Quality control of RNAs and microarrays for sorted DCs. Uninfected BMDCs (ctrl), or BMDCs infected with non-opsonized *L. am* amastigotes (*L. am*), Ab-opsonized *L. am* amastigotes (*L. am*-IS) were detached, stained with PE-CY5-conjugated anti-MHC II mAb and sorted without fixation by a high-speed sorting procedure. The sorting was performed with a FACSAria in a BSL-2 containment (*n* = 3 independent experiments). **(A)** Evaluation of RNA integrity after electrophoresis using Agilent Lab-on-chips. Total RNA extracted from sorted infected DCs and control uninfected DCs were analyzed. RNA integrity numbers (RIN) are indicated for representative samples. **(B)** Modulation of DC gene expression by *Leishmania* amastigotes. Differentially expressed probe-sets (adjusted *p* < 0.05) between sorted non-opsonized *L. am*-infected DCs and control cells are represented in red. Ab-opsonized amastigotes (*L.am*-IS) induce a much stronger effect than non-opsonized ones (*L.am*).

Supplementary Figure 5 Gene expression map of the NF-κB pathway in DCs infected with non-opsonized amastigotes of *L. amazonensis*. Significant modulations calculated between *L.am*-infected and uninfected BMDCs are represented by the color code, with red indicating up-regulated (linear FC >+1.5) and green down-regulated (linear FC < -1.5) genes. Symbols, lines and color codes are defined in the legend. White (left) and shaded (right) areas correspond to classical and alternative NF-κB pathways, respectively.

Supplementary Figure 6 STRING network analysis for modulated genes of transcription-related factors (TRFs) in DCs infected with non-opsonized amastigotes. Networks for down-modulated (green) **(A)** and up-modulated genes (red) **(B)** are shown.
