# Supplementary figures and images for: Leishmania amazonensis Subverts the Transcription Factor Landscape in Dendritic Cells to Avoid Inflammasome Activation and Stall Maturation
Source: Front Immunol. 2020 Jun 9;11:1098. doi: 10.3389/fimmu.2020.01098 (PMC7295916; doi:10.3389/fimmu.2020.01098)

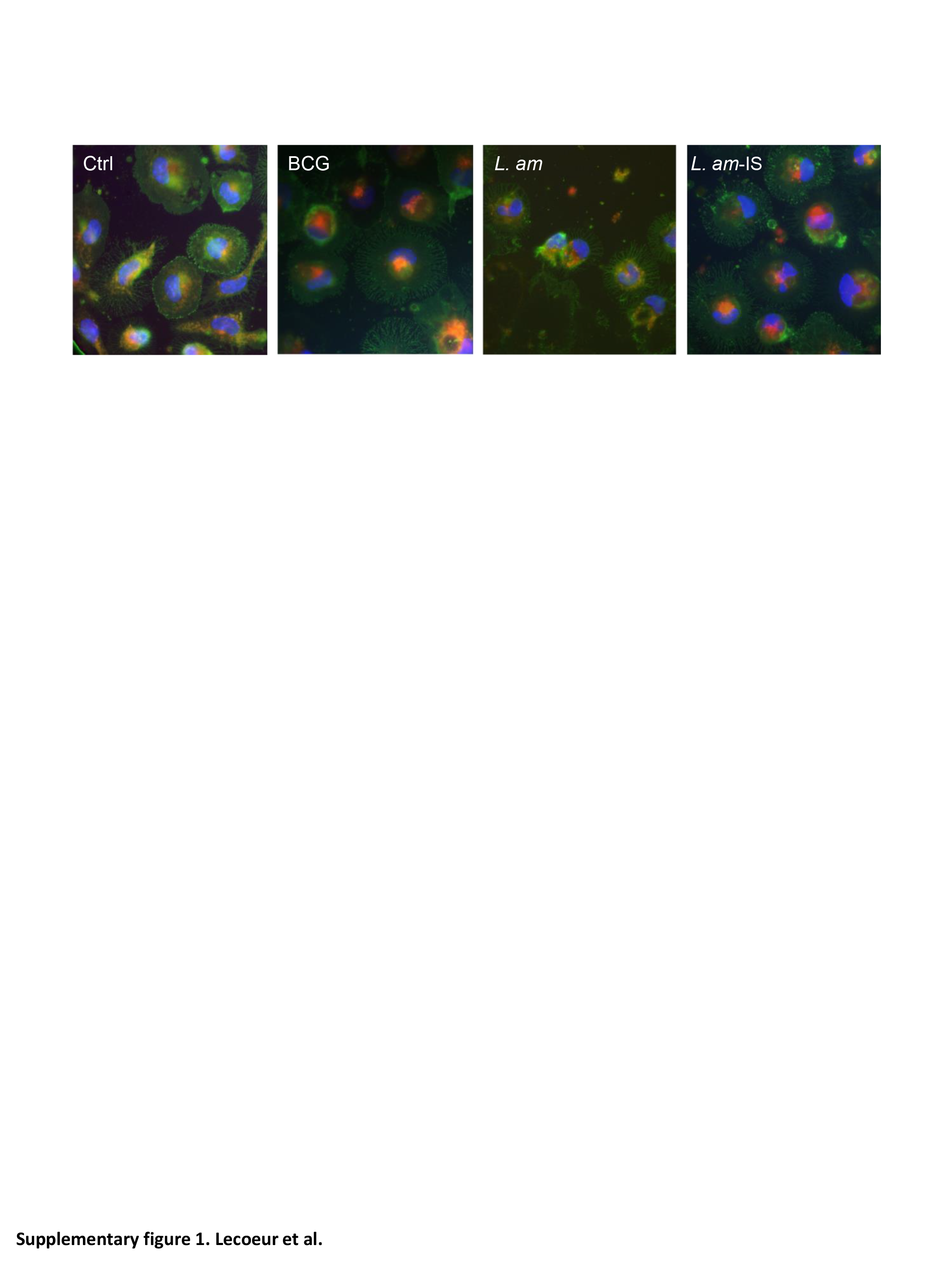

Supplement: Supplementary Figure 1 — Evaluation of the maturation status of DC cultures by epifluorescence microscopy. BALB/c-derived BMDCs alone (Ctrl) or incubated for 24 h with BCG (BCG) or L. amazonensis amastigotes without (L. am) or with Ab-opsonized (L. am-IS) were subjected to epifluorescence microscopy imaging of MHC Class II and H2-M molecule staining. Representative DCs from the different conditions are displayed as merged images of the 3 detection channels showing MHC II molecules (green), H2-M molecules (red), and nuclear DNA (blue). [file Image_1.TIF]

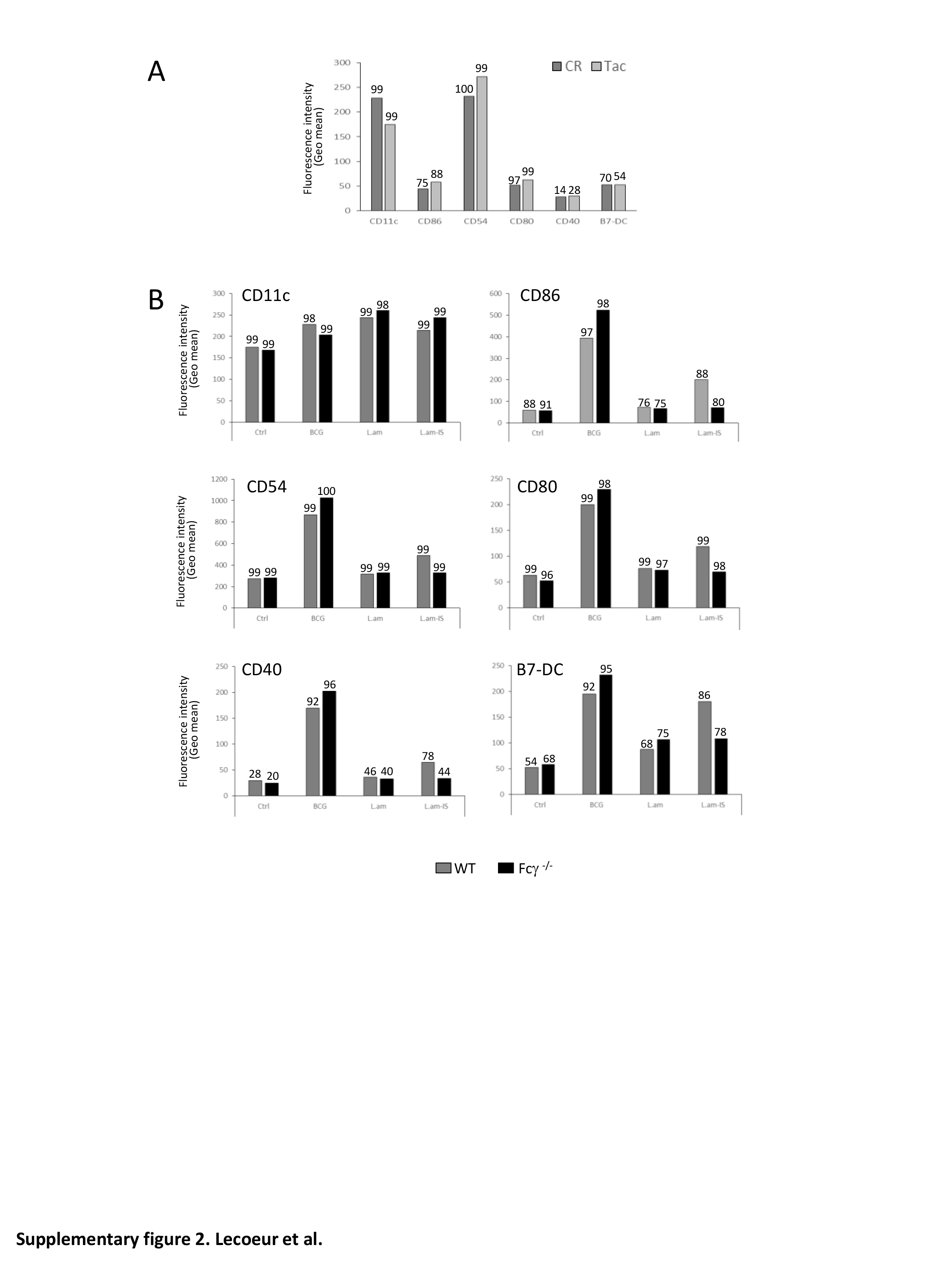

Supplement: Supplementary Figure 2 — Influence of the γ chain subunit of FcgammaRI, FcgammaRIII, and Fcepsilon RI receptors on the phenotype of DC cultures. (A) Flow cytometric comparison of surface marker expression of BMDCs from wild type mice from Charles River (CR) and Taconic (Tac) laboratories. Histograms show the mean fluorescence intensity of the indicated markers. Numbers represent the percentage of DCs expressing the corresponding marker. Note the similarity of the profiles between both sources of BALB/c mice. (B) Phenotypic analysis of DCs derived from wild type (gray histograms) and Fcγ−/− (black histograms) mice from Taconic laboratories. DCs alone (Ctrl) or incubated for 24 h with BCG or L. amazonensis amastigotes without (L. am) or with Ab-opsonization (L. am-IS) are shown. Histograms correspond to the mean fluorescence intensity of the indicated markers. Numbers represent the percentage of DCs expressing the corresponding marker. Note that the absence of the γ chain abrogates the marker increase observed in L. am-IS-infected DCs from WT background. [file Image_2.TIF]

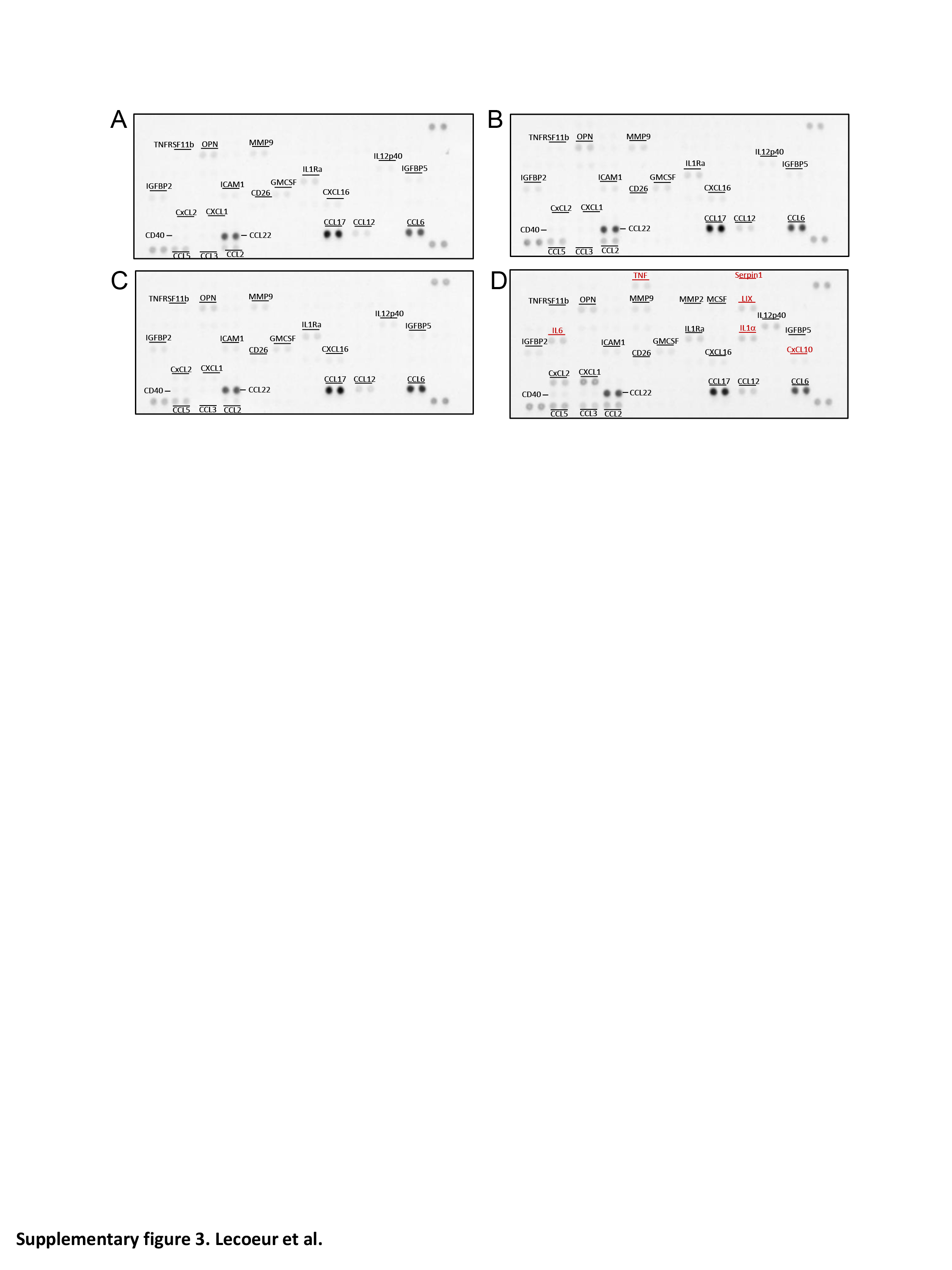

Supplement: Supplementary Figure 3 — Results of cytokine array analysis of DC culture supernatants. Uninfected BMDCs (ctrl, A), or BMDCs infected with non-opsonized L. am amastigotes (B), Ab-opsonized L. am amastigotes (C) or live BCG (D) are shown. Supernatants were analyzed using the mouse XL cytokine array kit. Pictures of the membranes are shown after revelation and scanning. Cytokines and chemokines already expressed in control cultures are labeled in black. Those specifically detected in the BCG-treated culture are labeled in red. [file Image_3.TIF]

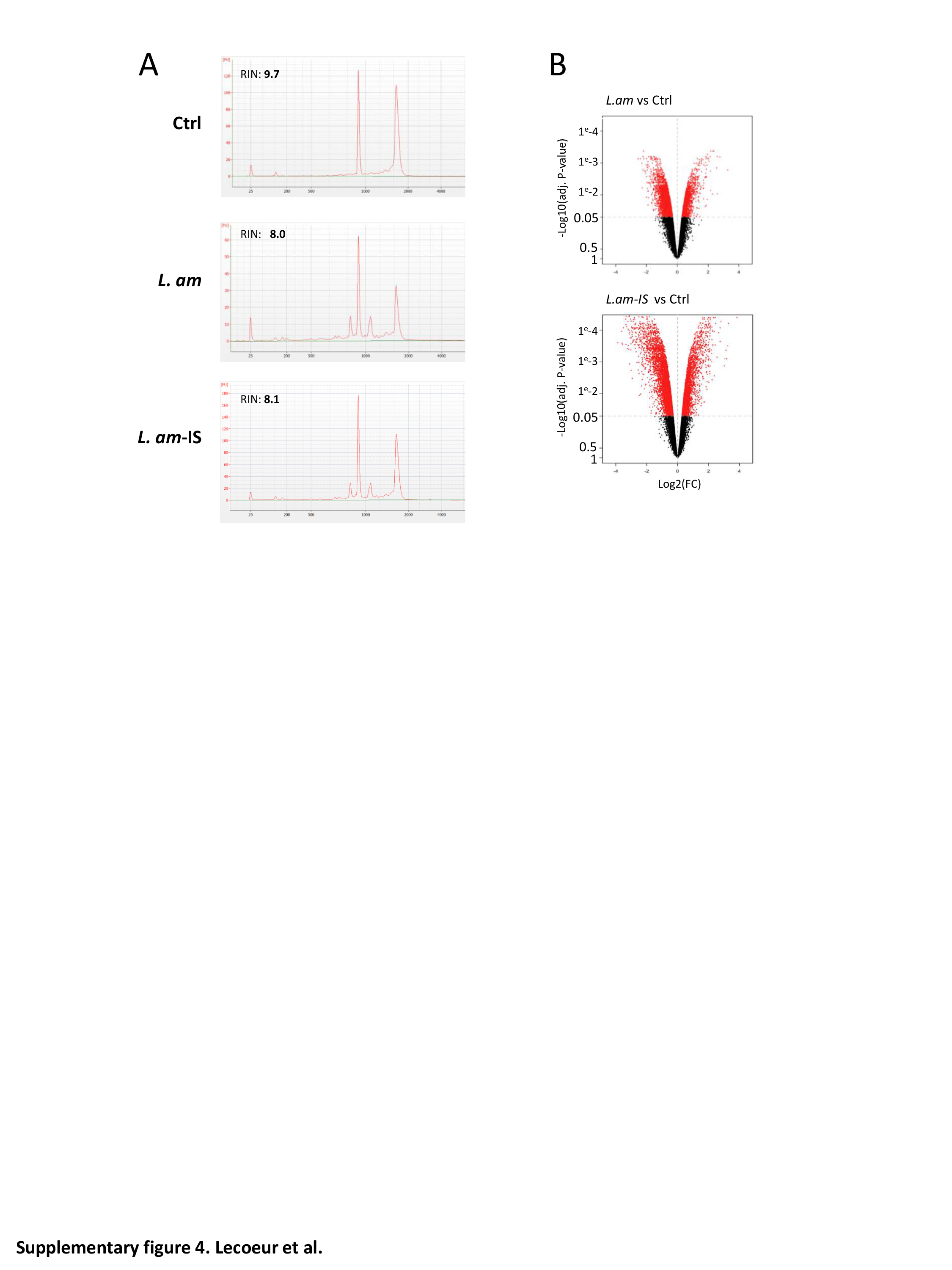

Supplement: Supplementary Figure 4 — Quality control of RNAs and microarrays for sorted DCs. Uninfected BMDCs (ctrl), or BMDCs infected with non-opsonized L. am amastigotes (L. am), Ab-opsonized L. am amastigotes (L. am-IS) were detached, stained with PE-CY5-conjugated anti-MHC II mAb and sorted without fixation by a high-speed sorting procedure. The sorting was performed with a FACSAria in a BSL-2 containment (n = 3 independent experiments). (A) Evaluation of RNA integrity after electrophoresis using Agilent Lab-on-chips. Total RNA extracted from sorted infected DCs and control uninfected DCs were analyzed. RNA integrity numbers (RIN) are indicated for representative samples. (B) Modulation of DC gene expression by Leishmania amastigotes. Differentially expressed probe-sets (adjusted p < 0.05) between sorted non-opsonized L. am-infected DCs and control cells are represented in red. Ab-opsonized amastigotes (L.am-IS) induce a much stronger effect than non-opsonized ones (L.am). [file Image_4.TIF]

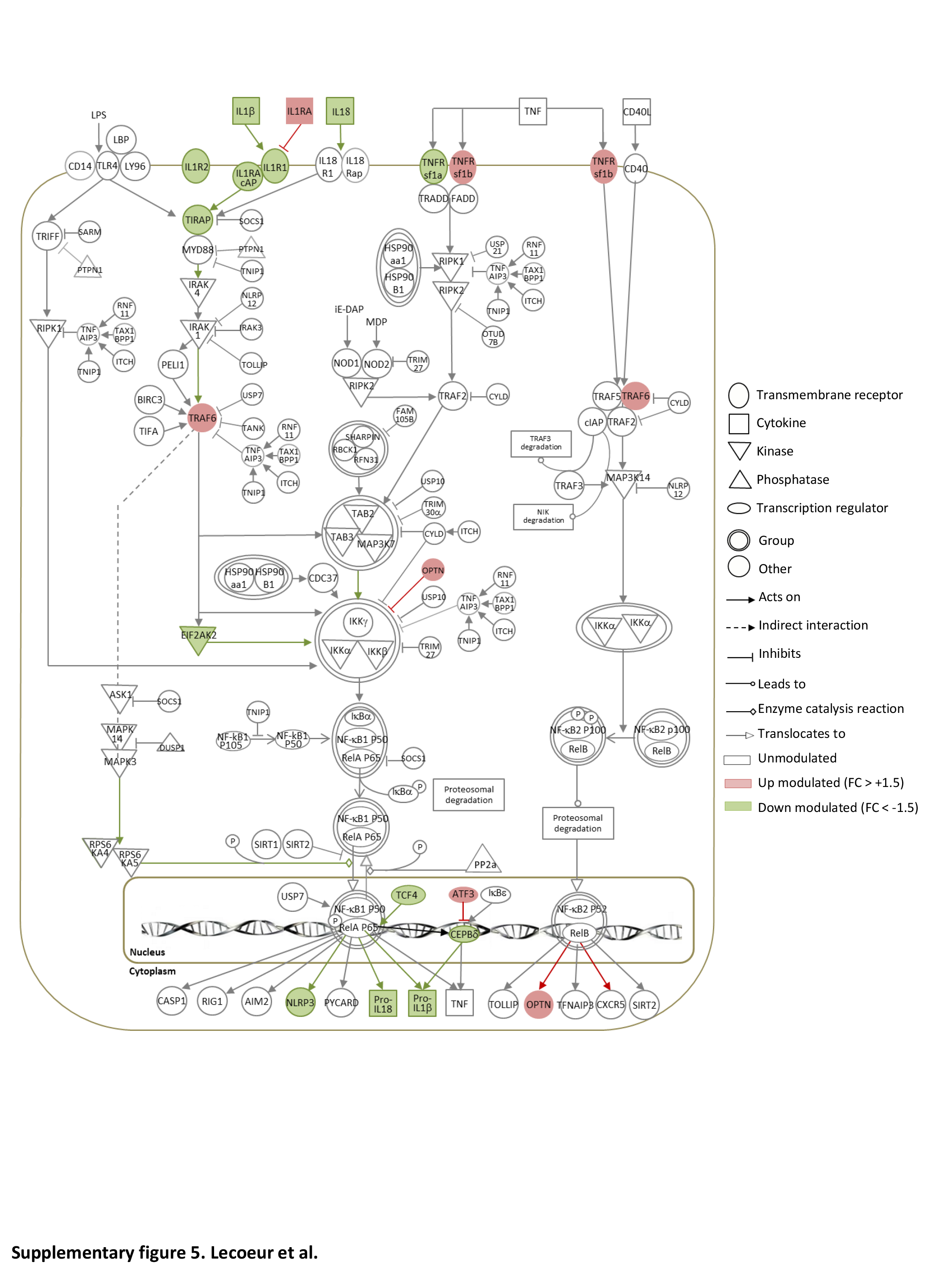

Supplement: Supplementary Figure 5 — Gene expression map of the NF-κB pathway in DCs infected with non-opsonized amastigotes of L. amazonensis. Significant modulations calculated between L.am-infected and uninfected BMDCs are represented by the color code, with red indicating up-regulated (linear FC >+1.5) and green down-regulated (linear FC < -1.5) genes. Symbols, lines and color codes are defined in the legend. White (left) and shaded (right) areas correspond to classical and alternative NF-κB pathways, respectively. [file Image_5.TIF]

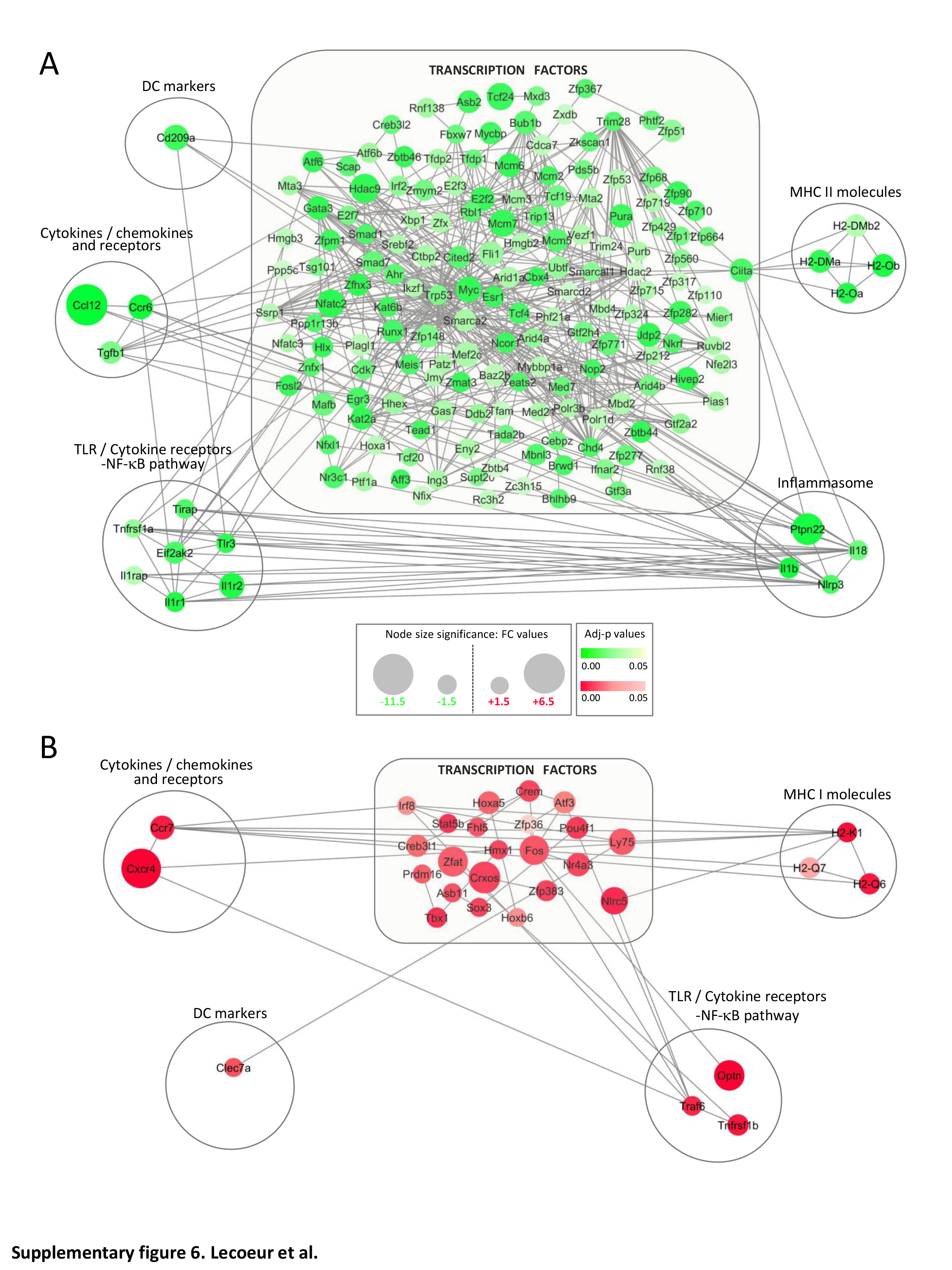

Supplement: Supplementary Figure 6 — STRING network analysis for modulated genes of transcription-related factors (TRFs) in DCs infected with non-opsonized amastigotes. Networks for down-modulated (green) (A) and up-modulated genes (red) (B) are shown. [file Image_6.TIF]
